# Comprehensive competency assessment of malaria microscopists and laboratory diagnostic service capacity in districts stratified for malaria elimination in Ethiopia​

**DOI:** 10.1371/journal.pone.0235151

**Published:** 2020-06-25

**Authors:** Desalegn Nega, Abnet Abebe, Adugna Abera, Bokretsion Gidey, Abeba G/Tsadik, Geremew Tasew

**Affiliations:** Ethiopian Public Health Institute, Addis Ababa, Ethiopia; Ministry of Health and Sports, MYANMAR

## Abstract

**Background:**

Federal Ministry of Health (FMoH) Ethiopia achieved significant declines in malaria mortality and incidence and has recently launched malaria elimination in selected low transmission settings. Successful malaria elimination calls for rapid and accurate diagnosis of cases so that the patients can promptly be treated before the occurrence of transmission. Therefore, this study assessed the competency of malaria microscopists using panal slides, and laboratory service availability and readiness in terms of supplies and equipments in malaria elimination targeted districts in Ethiopia.

**Method:**

A cross-sectional study was conducted from February to June 2018 in all hospitals, health centers and private clinics in 20 malaria elimination targeted districts, selected out of the 6 regional states in Ethiopia. All malaria microscopists available in the study health facilities during the study period were included in the study. Questionnaires were used for interviewing sociodemography of personnel and laboratory supplies. Per World Health Organization (WHO) criteria set for proficiency testing, 10 Giemsa stained malaria slide panels (8 positive low/high density pf/pv/Mixed and 2 negative slides) were administered to each study participant for performance assessment on malaria parasite detection, species identification and parasite count using light microscopy. The slide panels are PCR confirmed and WHO approved ones, which have been stored in the slide banks at the national reference laboratory in Ethiopian Public Health Institute.

**Result:**

In this assessment, 17(16%) district hospitals, 71(67%) health centers (HCs) and 18(17%) private clinics (PCs) were included. Of the 18 PCs, only 10(55.6%) had license certificate. Of the study facilities, 91.5%(97) use light microscopy, 2.83%(3) use RDTs and 2.9%(3) use both microscopy and RDT to detect malaria. Accessible and appropriate storage of Giemsa was reported by 58.8%(10) hospitals, 81.7%(58) HCs & 72.2%(13) private clinics. Of the 1896 malaria positive & 474 negative slides administered to 237 study participants, 318(16.8%) slides reported falsely negative & 47(9.9%) reported falsely positive. The participants achieved “*good”* grade [Agreement(A): 84.6%, Kappa(K): 0.6] on parasite detection and “*poor”* agreement (A: 43.8%; K: 0.11) on every species identification. No or slight agreement seen on differentiation of *P*. *falciparum* from other species (A: 28.41%; K:0.29). Above 95%(201) of participants, did not count or used plus system of parasite estimation which is the least accurate and unreccomended method per WHO guideline.

**Conclusion:**

In the current study, low performance of malaria microscopists particularly in species identification & poor to moderate capacity of laboratories observed. This is really a great obstacle to malaria elimination strategy of the country. Therefore, national malaria control and elimination program in collaboration with partners is supposed to provide comprehensive training for professionals giving laboratory service and to fulfill laboratory supplies to have the gold standard service.

## Introduction

In Ethiopia, About 75% of the land mass is malarious and the proportion of the population at risk of malaria is about 60% with 54 (6.4%) woredas having high transmission, mainly at altitudes below 2,000 meters. There was a reduction in malaria prevalence by microscopy in 2015 (0.5 percent) compared to the results in 2011 (1.3 percent). Similarly, when comparing the Rapid Diagnostic Test (RDT) results, malaria prevalence in 2015 (1.2 percent) was reduced compared to that of 2011 results (4.5 percent) [1]. The pooled prevalence of *P*. *falciparum* and *P*. *vivax* parasites estimates in a proportion of 62.8% and 37.2%, respectively [2]. There are different malaria diagnostic methods for malaria case management; the common and routine ones are patient‘s clinical assessment, microscopic examination of blood slides and the use of rapid diagnostic tests (RDTs). The detection of *Plasmodium* parasites by light microscopy remains the backbone of parasite-based diagnosis in most health care facilities throughout the world [3,4]. It has long been the method of choice for the diagnosis of parasitic diseases, and is the gold standard tool for routine malaria diagnosis still now [4–7].

Definitive diagnosis based on clinical manifestations is not possible; because many of the signs and symptoms overlap with that of other febrile illnesses. Accordingly, the Ethiopian malaria national strategic plan states that 100% of suspected cases should be diagnosed in the laboratory within 24 h of fever onset [8]. Lack of qualified professionals in malaria diagnosis and the lack of regular quality control approaches in the laboratory diagnostic process have been identified as the main reasons for the lack of success in the current strategy to control malaria [9]. Low performance of professionals in microscopic diagnosis of malaria is a great challenge, which leads to inappropriate and/or delayed treatment, emergence of drug resistance, development of serious complications up to the death of patients [10]. Major challenges in laboratory capacity include poor physical infrastructure and inadequate supplies such as equipments and reagents, limited human capacity, lack of clear laboratory policies and strategic plans, and limited collaborations between clinical and laboratory services [11,12].

The main goals of the current malaria National Strategic Plan (NSP) 2014–2020 Ethiopia [13] are: i) to achieve near zero malaria deaths (no more than 1 confirmed malaria death per 100,000 population at risk) in Ethiopia by 2020; ii) to reduce malaria cases by 75% from baseline of 2013 by 2020; iii) to eliminate malaria in selected low transmission areas by 2020 and nationwide by 2030. Decrease in malaria burden in Ethiopia is not uniform at whole geographies; hence, the country planned to eliminate malaria with a stepwise approach by primarily targeting the low-transmission districts and their adjacent areas/zones in order to shrink the country’s malaria map progressively. Elimination program classifies districts into different phases depending on the level of annual parasite incidence (API): Phase 1: Optimization (API of 5–10); 2) Pre-elimination (API of 1–5); 3) Elimination (API of <1); 4) Prevention of re-introduction (API of zero) [14].

Districts graduate from the optimization phase and enter pre-elimination when their API is less than five, and districts graduate from the pre-elimination phase and enter elimination with an API less than 1. In optimization phase, the objective is to maximize existing interventions and reduce the burden of malaria to low levels. In contrast, the purpose of pre-elimination phase is to further reduce transmission and introduce additional approaches and tools to the existing optimization interventions. The elimination phase focuses on the interruption of local transmission or ending local transmission. This is achieved by detecting every infection and managing each case properly [14].

In Ethiopia, malaria diagnosis in hospitals, health centers and in most private clinics relies dominantly on Giemsa smear microscopy. Ethiopia scaled up diagnostic testing for malaria at all levels of the public health sectors: multispecies RDTs are used at community-level in health posts and malaria microscopy is carried out at district-level health centers as well as district, zonal and regional-level hospitals [15]. Early diagnosis and a prompt treatment are the key strategies for the management of malaria to reduce mortality and morbidity in Ethiopia [4,16,17].

To achieve these goals, the country must properly and consistently implement policies and practices that improve the quality of confirmatory laboratory diagnosis of malaria. Except limited pocket studies, a comprehensive assessment of the proficiency of malaria microscopists and laboratory capacity in terms of equipments, reagents and laboratory setup has never been done in Ethiopia. High quality malaria diagnostics is acknowledged as a crucial element of successful malaria elimination [18,19], with microscopy as the gold standard playing a key role [19,20]. Experience from malaria-free countries has demonstrated the importance of well-established, widespread, high quality malaria diagnosis, high capacity for active case follow-up and a robust monitoring and evaluation system [21–23].

One of the most important approaches for quality assurance of malaria diagnosis is regular competency assessment of microscopists, and assessments of gaps and shortages in laboratory capacity in terms of equipments, reagents, supplies and even the lab set up. Hence, this study was conducted to assess the competency of malaria microscopists, and laboratory diagnostic service capacity in districts stratified for malaria elimination in Ethiopia.

## Materials and methods

### Study setting and period

**Fig 1:**
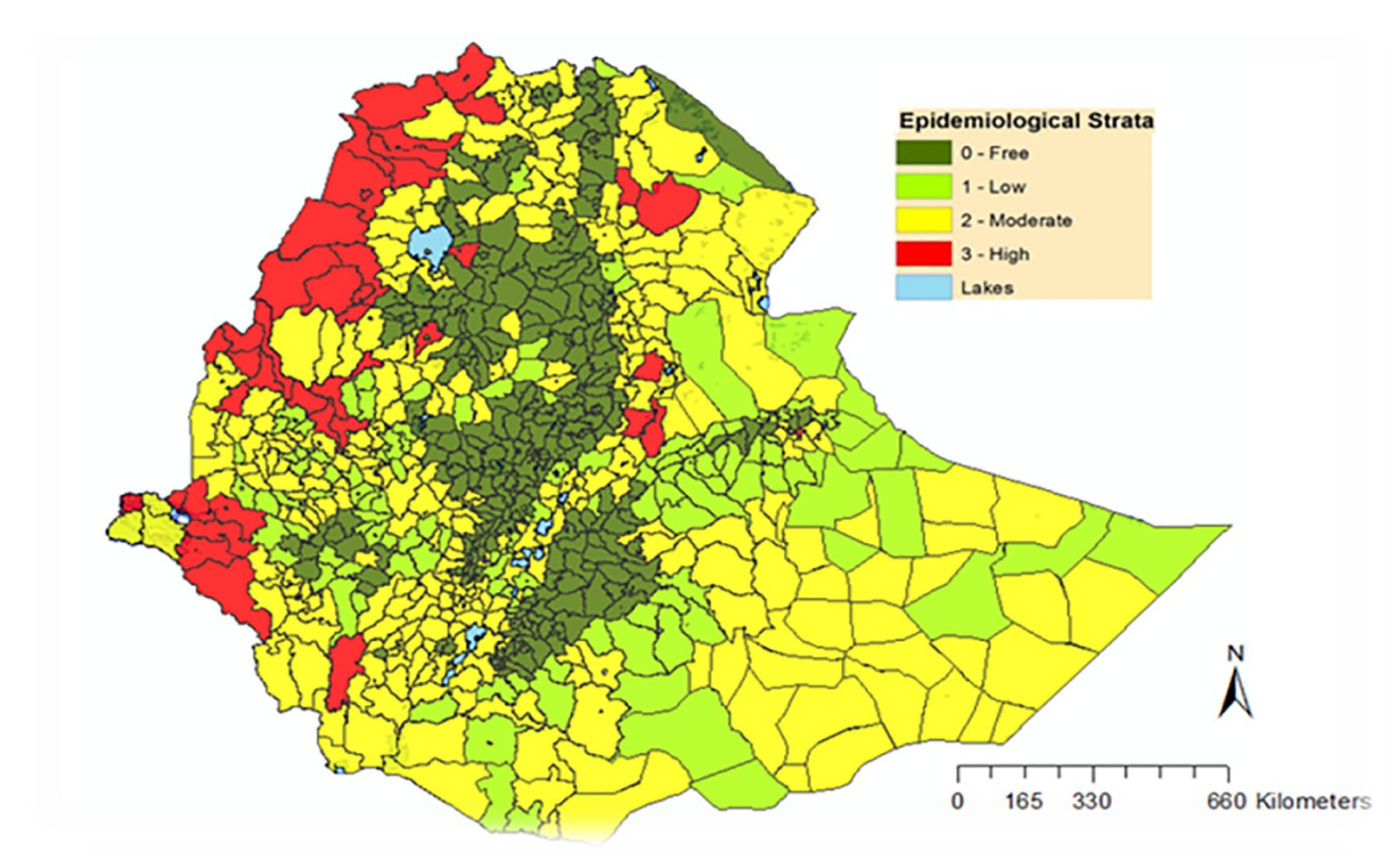
Malaria risk map of districts by annual parasite incidence, Ethiopia (Source FMOH NSP: 2017–2020).

The study was conducted from February to June 2018 in malaria elimination targeted districts (equally called as Woredas in local language) in Ethiopia. The country currently launched malaria elimination in selected 239 districts with low malaria transmission (**Fig 1**); these districts were located in Dire Dawa City Administration, Harari region, Oromia region, Tigray region and Southern (SNNP) regional states. From the 239 malaria elimination targeted districts, 20 districts with high API were selected for this study.

### Study design

A descriptive cross-sectional study design was used. Twenty districts reporting substantial malaria cases were purposely selected from among the 239 malaria elimination targeted districts in the country. All hospitals, health centers, and private clinics in the districts were included. Only facilities with out functional laboratory because of lack of microscope or no laboratory technician/ologist were not enrolled.

### Study subjects and inclusion

All malaria microscopists available at the diagnostic facilities during data collection period were included. Professionals on leave due to sickness & maternal cases, and the non-consenting ones were excluded from study.

### Sample size determination

A maximum of five malaria-microscopists per facility, available in the facility during the study period, were conveniently included in the study.

### Sampling technique

Convenient sampling (consecutive enrollment) method was used until we had enrolled the eligible professionals in each laboratory.

### Data collection tools

#### Malaria slide panels

Slide panels were used to test the competecy of malaria microscopists on parasite detection, species identification and parasite count. Standardized malaria slide panels were distributed as the following (Table 1).

**Table 1:** Composition of blood film slide for examining study partciparts.

S.N	Composition of Blood Film(BF) slides (Species)	Number of BF slides	Parasites Densities
01	*P*.*falciparum* of low densities	2	343
			1817
02	*P*.*falciparum* of high densities	1	66,973
03	*P*.*vivax* of low densities	2	1,390
04	*P*.*vivax* of high densities	1	49,372
05	Mixed (*P f+ Pv*) of low densities	1	20,711
06	Mixed (*P f+ Pv*) of high densities	1	89,500
07	Negative blood film slides	2	Not applicable
	Total	10	

#### Study questionnaires

Structured questionnaires including information on the participating facilities, laboratory capacity (equipment, reagents and laboratory setup) and socio-demography of professionals were used.

### Data collection process

A total of 10 Giemsa-stained malaria slide panels (with different species and densities) from the national malaria slide bank were administered to study participants for the performance assessment on parasite detection, species identification and quantification. Ten minutes per slide were allocated to each participant to examine the blood film slides. At the same time, questionnaires with closed and open ended questions were utilized to interview the sociodemography of the laboratory professionals and to query the laboratory in terms of supplies and equipments. When the microscopists had completed reading the slide panels in the given time, the slides were collected back to their box for administration in other facilities when the data collection teams were moving.

### Statistical analysis

Data was entered into and analyzed using statistical packages for windows (SPSS 20). Descriptive statistics was used to calculate proportions, frequencies, ratios, ranges and others. Based on the calculation, the strength of agreement between participants and WHO-certified expert was classified as Kappa value: 0.01–0.20 slight agreement, 0.21–0.40 fair agreement, 0.41–0.60 moderate agreement, 0.61–0.80 substantial agreement, and 0.81–1.00 almost perfect agreement [24,25].

### Ethical consideration

The research proposal was ethically cleared by the Scientific and Ethical Review Committee (SERO) of the Ethiopian Public Health Institute (EPHI). Official cooperation letters were written by EPHI to the participating facilities. Consent forms were used to take the agreement of participating health professionals. To ensure confidentiality, the participants’ data were linked to a specific code number only.

## Results

### Socio demographic characteristics of the study participants

The mean age of the study participants was 28.9 years (28 years and 9 months) (Range: 20–52 years). Most of the participants were in the age group of 20–30 years. Those who held diploma certificate accounted 68.1%, bachelor degree 31.5%, and master degree 0.4%. Most microscopists (62.2%) were graduates of government college. Around 40% of microscopists did not have refresher training on malaria microscopy within 2 years prior to study commencement **(Table 2).**

**Table 2:** Socio demographic characteristic of microscopists in districts stratified for malaria elimination in Ethiopia.

Characteristic		Frequency	Percent (%)
**Age(Years)**	20–30	179	75.2
	31–40	51	21.4
	≥41	8	3.4
**Sex**	Male	150	63.0
	Female	88	37.0
**Type of college**	Government	148	62.2
	Private	90	37.8
**Level of Education**	Diploma	162	68.1
	BSc	75	31.5
	MSc	1	0.4
**Work Experience (in Years)**	< 2	39	18.3
	2–5	66	31.0
	>5	108	50.7
**In-service Training with the past 2 yrs**	Trained	140	59.6
	Not trained	95	40.4
**Training Provided by**	Government	114	82.0
	Partners	25	18.0
**Place of Work**	Public (Government)	217	91.2
	Private	21	8.8

#### Laboratory infrastructure and services

This assessment was conducted in total of 106 health facilities: 17 district hospitals, 71 health centers and 18 private health facilities. Relatively, hospitals had good bench space, sink, washing and staining area, ventilation and lab space, and disposal of waste materials per national guideline compared to health centers and private clinics. Storage space for supplies & materials was observed poor in 2(13.3%) hospitals, 24(33.8%) health centers and 8(47.1%) private clinics. Of the 18 private clinics, only 10(55.6%) had license & registration certificate. Of the facilities, 98(92.4%) use only light microscopy, 4(3.8%) use only malaria RDTs and 3.8%(4) use both microscopy and RDT to detect malaria. Among the health facilities, 29(28.7%) facilities were examining less than five slides per day, 29(28.7%) were examining 5–10 slides/day and 43(42.6%) facilities were examining more than 10 slides/day. Accessible and appropriate storage of Giemsa was reported by 14(83.3%) hospitals, 62(87.3%) health centers & 15(83.3%) private clinics. Out of the total health facilities, 56(54.9%) were regularly being supervised by regional and national reference laboratories within one year prior to study period (Table 3).

**Table 3:** General information on laboratory services in districts stratified for malaria elimination in Ethiopia.

Variables	Response Category	District Hospital	Health centre	Private hospital/clinic	Total
Types of health facility, n(%)		17(16)	71(61)	18(17)	106(100)
Registered and licensed private laboratory	Yes	NA	NA	10(55.6)	10(55.6)
	No	NA	NA	8(44.4)	8(44.4)
Supervision from regional or national laboratories within a year	Yes	12(80)	37(52.9)	7(41.2)	56(54.9)
	No	3(20)	33(47.1)	10(58.8)	46(45.1)
Method of diagnosis for malaria	Microscope	16(94.1)	66(93.0)	16(88.9)	98(92.4)
	RDT	1(5.9)	2(2.8)	1(5.6)	4(3.8)
	Both	0	3(4.2)	1(5.5)	4(3.8)
Daily malaria slides examined	Less than 5	1(6.2)	22(31.9)	6(37.5)	29(28.7)
	From 5–10	2(12.5)	21(30.4)	6(37.5)	29(28.7)
	More than 10	13(81.2)	26(37.7)	4(25)	43(42.6)

#### Laboratory technical activities and programmatic issues

Functional microscope was reported available in all 17 study hospitals, in 67(94.4%) of health centers and 13(81.2%) of private clinics during the survey. In this study, above 95% of health facilities use the plus system of parasite density estimation. Regular external training program for microscopists was reported available in 12(12.9%) facilities while the remaining had none. Out of the total health facilities, 51(50.5%) were not participating in any of the external quality assessment (EQA) schemes (Table 4).

**Table 4:** General information on technical and programmatic issues in districts stratified for malaria elimination in Ethiopia.

Variables	Response Category	District Hospital	Health centre	Private hospital/clinic	Total
**Functional microscope available**	Yes	16(100)	67(94.4)	13(81.2)	96(93.2)
	No	0	4(5.6)	3(18.8)	7(6.8)
**Malaria blood smear preparation**	Thin only	0	1(1.4)	0	1(1)
	Thick only	4(25)	19(26.8)	7(43.8)	30(29.1)
	Thick and thin	12(75)	51(71.8)	9(65.2)	72(69.9)
**Perform parasite count**	Yes	10(62.5)	24(35.3)	8(50)	42(42)
	No	6(37.5)	44(64.7)	8(50)	58(58)
**Parasite count types used**	Plus system	11(100)	25(92.6)	11(100)	47(95.9)
	Parasite/μl/WBC	0	1(3.7)	0	1(2)
	Parasite/μl/RBC	0	1(3.7)	0	1(2)
**Regular training program for microscopists available**	Yes	3(23.1)	9(13.6)	0	12(12.9)
	No	10(76.9)	57(86.4)	14(100)	81(87.1)
**Participate in any EQA programs**	Yes	9(52.9)	37(52.1)	4(23.5)	50(49.5)
	No	6(47.1)	32(47.9)	13(76.5)	51(50.5)

#### Laboratory documents and records

Absence of standard laboratory request form was reported by 6(37.5%) of hospitals, 44(63.8%) of health centers and 11(64.7%) private clinics. Results were recorded legibly in logbooks of 14(87.5%) hospitals, 61(87.1%) health centers, and 75(87.2%) private clinics. Standard operating procedures were reported absent at 2(12.5%) hospitals, 30(42.9%) health centers and 12(70.6%) private clinics. Technical manuals and lab bench aids were observed in 9(56.2%) hospitals, 40(57.1%) health centers and 6(35.3%) private clinics. Internal quality control log sheet was reported unavailable in 3(18.8%) hospitals, 39(55.7%) health centers and 15(88.2%) private clinics. In 11(84.6%) hospitals, 25(35.7%) health centers & 4(26.7%) private clinics, the laboratories complied fully with the national quality assurance guidelines. Internal quality control was not being performed in 6(37.5%) hospitals, 42(61.8%) health centers, and 13(86.7%) private clinics. Buffered distilled water was used to dilute Giemsa stain in only 17(16.7%) facilities whereas 87(83.3%) facilities were using tap water (Table 5).

**Table 5:** Laboratory documents and records, in districts stratified for malaria elimination in Ethiopia.

Variables	Response Category	District Hospital	Health Centre	Private Hospital/Clinic	Total
Availability of standard laboratory request form	Yes	10(62.5)	25(36.2)	6(35.3)	41(40.2)
	No	6(37.5)	44(63.8)	11(64.7)	61(59.8)
Organized result recording in logbooks	Yes	14(87.5)	61(87.1)	10(58.8)	85(82.5)
	No	2(12.5)	9(12.9)	7(41.2)	18(17.5)
SOPs availability in laboratory	Yes	14(87.5)	40(57.1)	5(29.4)	59(57.3)
	No	2(12.5)	30(42.9)	12(70.6)	44(42.7)
Technical manuals and bench aids in the laboratory	Yes	9(56.2)	40(57.1)	6(35.3)	55(53.4)
	No	7(43.8)	30(42.9)	11(64.7)	48(46.6)
Internal quality control log sheet available	Yes	13(81.2)	31(44.3)	2(11.8)	46(44.7)
	No	3(18.8)	39(55.7)	15(88.2)	57(55.3)
Maintenance logbook for microscope	Yes	14(87.5)	41(59.4)	4(23.5)	59(57.8)
	No	2(12.5)	28(40.6)	13(76.5)	43(42.2)

#### Laboratory supplies and equipment

All participant laboratories were assessed for their laboratory supplies and equipment status in terms of quality and quantity. From the study health facilities, 63.5% of governmental facilities and 52.9% of private health facilities were not conducting regular microscope maintenance services like cleaning surface of microscope components, checking all optics for damage, removing oil from objective and replace bulbs as needed (Table 6).

**Table 6:** Laboratory supplies and equipment over the last six months prior to data collection in districts stratified for malaria elimination in Ethiopia.

Supplies and Equipment	Response Category	Government Health Facilities (n%)			Private Clinic (n%)	Total (n%)
		District Hospitals	Health center	Total		
**Microscope binocular with x100 objective**	Yes	14(93.3%)	70(98.6%)	84(97.7)	16(100.0%)	100(98.0%)
	No	1(6.7)	1(1.4)	2(2.3)	0	2(2)
**Regular microscope maintenance service**	Yes	6(42.9)	25(35.2)	31(36.5)	8(47.1)	39(38.2)
	No	8(57.1)	46(64.8)	54(63.5)	9(52.9)	63(61.8)
**Availability of spare bulbs for microscope**	Yes	9(69.2)	18(25.2)	27(32.5)	5(29.4)	32(32)
	No	4(30.8)	52(74.3)	56(67.5)	12(70.6)	68(68)
**Availability of quality and cleaned microscope slides**	Yes	13(86.7)	62(87.3)	75(87.2)	12(70.6)	87(84.5)
	No	2(13.3)	9(12.7)	11(12.8)	5(29.4)	16(15.5)
**Re-using microscope slides**	Yes	6(40)	18(25.4)	24(27.9)	9(52.9)	33(32)
	No	9(60)	53(74.6)	62(72.1)	8(47.1)	70(68)
**Availability of all required reagents**	Yes	14(93.3)	59(84.3)	73(85.9)	7(41.2)	80(78.4)
	No	1(6.7)	11(15.7)	12(14.1)	10(58.8)	22(21.6)
**Storage of staining solutions per the manufacturers guide**	Yes	13(92.9)	53(77.9)	66(80.5)	13(81.2)	79(80.6)
	No	1(7.1)	15(22.1)	16(19.5)	3(18.8)	19(19.4)

#### Performance of participants in detection and identification of malaria parasites

Competency in microscopy is the ability of a microscopist to examine malaria blood film and report the results accurately. WHO Malaria microscopy quality assurance manual version 2 clearly defines on how to grade the performance of parasite detection and species identification. Of 1896 malaria positive & 474 negative slides administered to 237 participants, 318 slides(16.8%) reported falsely negative & 47(9.9%) reported falsely positive (Table 7).

**Table 7:** Performance of participants on parasite detection in districts stratified for malaria elimination in Ethiopia.

Crosstab		Expert parasite detection			Sensitivity	Specificity	Agreement (A)
		Positive	Negative	Total	83.2%	90.1%	84.6% [Kappa(K) = 0.6]
**Participant parasite detection**	Positive	1578	47	1625			
	Negative	318	427	745			
	Total	1896	474	2370

- Percentage of slides in agreement in detection, i.e. percentage of *positive* slides correctly identified and percentage of *negative* slides correctly identified:
  $$\%\mathrm{Agreement}=\frac{\mathrm{TP}+\mathrm{TN}}{\mathrm{TN}+\mathrm{TP}+\mathrm{FN}+\mathrm{FP}}x100\%=\frac{1578+427}{2370}x100\%=84.6\%;\;\mathrm{Cohen}’s\;k:\;0.6;$$
  This is a good grade in parasite detection (Range: 75%≤85%).
- Sensitivity: Proportion of positive slides correctly read as positive
  $$\mathrm{Sensitivity}=\frac{\mathrm{TP}}{\mathrm{TP}+\mathrm{FN}}X100\%=\frac{1578}{1578+318}X100\%=83.2\%$$
- Specificity: Proportion of negative slides correctly read as negative
  $$\mathrm{Specificity}=\frac{\mathrm{TN}}{\mathrm{TN}+\mathrm{FP}}X100\%\frac{427}{427+47}X100\%=90.1\%$$

#### Performance of participants in the differentiation of Plasmodium species

Referring to expert readers, the participants achieved “*good”* grade (Agreement: 84.6%, Kappa: 0.6) on parasite detection and “*poor”* agreement (Agreement: 43.8%; Kappa: 0.11) on species identification. No or very slight agreement seen on differentiation of *P*. *falciparum* from other species (Agreement:28.41%; K: -0.29) (Table 8).

**Table 8:** Performance of participants in the differentiation of Plasmodium species in districts stratified for malaria elimination in Ethiopia.

Crosstab			Expert parasite species identification			Agreement (A)	Cohen’s k
			Single/mixed spp present	Negative	Total		
Participant species identification		Correct	508	47	555	43.8%	**K: 0.1099 Slight agreement**
		Incorrect	1151	427	1578		
		Total	1659	474	2133		
**The accuracy of the differentiation of *P. falciparum* from other species**							
			Pf only/mixed	Non-Pf	Total		
Participant species identification	Pf only/mixed		275	345	620	28.4%	**Cohen’s k: -0.2985 No agreement**
	Non-Pf		673	129	802		
	Total		948	474	1422		

#### Overall performance of malaria microscopists against sociodemography

The performance of participants with master degree have higher percent agreement on parasite detection than participants with bachelor degree and Diploma certificate. Graduates from government or public universities/colleges had better performance than graduates of private colleges. Participants skill also differs based on the experience years; the higher the service year the higher is participants capacity to detect and identify parasites (Table 9).

**Table 9:** Overall performance of malaria microscopists against sociodemography in districts stratified for malaria elimination in Ethiopia.

Variables	Degree	Result	Expert reading			Agreement b/n participant & expert	
			Parasite Detection	Species Identification	Negative	Parasite Detection	Species Identification
Level of Education	**Diploma**	*Pos*	1060	619	40	A: 82.96%	A: 61.93%
		*Neg*	236	515	284	K:0.57	K:0.28
	**BSc**	*Pos*	510	363	9	A: 86.8%	A: 74.7%
		*Neg*	90	162	141	K:0.66	K = 0.46
	**MSc**	*Pos*	8	5	0	A: 100%	A: 70%
		*Neg*	0	3	2	K:1	K: 0.39
Type of college	**Government**	*Pos*	1459	926	43	A: 85%	A: 67.4%
		*Neg*	277	593	391	K:0.62	K:0.35
	**Private**	*Pos*	119	61	6	A: 73.8%	A: 51.3%
		*Neg*	49	86	36	K:0.41	K:0.16
Work Experience	**< 2 yrs**	*Pos*	256	156	18	A: 81.0256%	A: 61.5384%
		*Neg*	56	117	60	k: 0.49	K: 0.23
	**2–5 yrs**	*Pos*	441	253	12	A: 85%	A: 62.79%
		*Neg*	87	209	120	k: 0.61	k: 0.29
	**>5 yrs**	*Pos*	719	464	16	A:85.09%	A: 68.31%
		*Neg*	145	292	200	K: 0.62	K:0.37
In-service Training	**Trained**	*Pos*	946	614	28	A: 85.57%	A:71.32%
		*Neg*	174	366	252	K: 0.62	K:0.44
	**Not trained**	*Pos*	610	357	19	A: 82.21%	A: 61.754%
		*Neg*	150	308	171	K:0.56	K:0.28

#### Performance of participants in the detection and identification of malaria parasites against different variables

On both parasite detection and species identification, the agreement is lower in private clinics (detection agreement: 77.8%; species identification agreement: 37.2%), followed by health centers (detection agreement: 84.14%; species identification agreement: 41.64%) and hospitals (detection agreement: 86.7%; species identification agreement: 48.1%) (Table 10).

**Table 10:** Overall performance of malaria microscopists in districts stratified for malaria elimination in Ethiopia.

		Expert reading			% Agreement	
	Result	Parasite detection	Species identification	Negatives	Parasite detection	Species identification
All study participants reading	Pos	1578	508	47	A: 84.6%; K = 0.6	A:43.8%; K: 0.11
	Neg	318	1151	427		
	Total	1896	1659	474		
Government Hospital staff	Pos	639	231	12	A:86.7; K: 0.65	A: 48.1; K:0.16
	Neg	113	427	176		
	Total					
Health centre staff	Pos	820	246	31	A: 84.14; K:0.588	A: 41.64; K:0.08
	Neg	164	615	215		
	Total					
Private facility staff	Pos	119	31	4	A: 77.8; K:0.478	A: 37.2; K:0.06
	Neg	41	109	36		
	Total	1896	1659	474		

#### Parasite counting system

About 230 (97.0%) of the participants used plus system of parasite estimation which is the least accurate and unreccomended method per WHO guideline. Others 7(3.5%) used parasite count against 8000 WBCs, 1(0.4%) participant from government hospital counted parasites per parasite/μl against RBC, whereas the remaining study participants 29(12.18%) completely did not count the parasites

## Discussion

Microscopic examination of Giemsa-stained thick and thin blood films is the gold standard method for diagnosis of malaria. High quality laboratory equipments and laboratory supplies are very important to provide accurate and reliable laboratory results for customers. A total of 106 health facilities laboratories were assessed for their laboratory supplies and equipments in terms of quality and quantity. Most of the supplies such as alcohol, cotton, lancets, Giemsa stain, disposables gloves, lens cleaning solution, sharp containers, staining rack, drying rack were reported sufficiently available at 73.3% of health facilities whereas these supplies were reported insufficient or not available at 26.7% health facilities. The reason for insufficient quantity or unavailability at all at some health facilities may be due to poor inventory system of the laboratories, the lack of finance to purchase the supplies on time, shortage of supplies when the donor terminate the support, lack of commitment from staffs to forecast the supplies on time and lack of follow up on the requested supplies, failure of governmental organizations to distribute supplies on time, or may be the short expiry date of the supplies.

Competency assessment is one of the methods to verify microscopists’ competency to perform laboratory tests and produce accurate, reliable and timely results. In the current study, an agreement between the participants and malaria microscopy expert readers in the detection of malaria parasites was 84.6% (K: 0.6) which is relatively lower when compared to findings of similar studies conducted in Ethiopia where an agreement was 96.8% (k: 0.9) [26], study conducted in Hawassa town, Ethiopia where an agreement was 88% (k: 0.67) [27] and study conducted in Bahirdar, Ethiopia where agreement was 88.5% (k: 0.78) [28]. The agreement in the current study was relatively higher than the study conducted in Tigray, Ethiopia which was 79% (kappa = 0.62) [29]. The reason for this variation may be due to difference in malaria endemicity which affects microscopists capacity of diagnosis and lack of regular training which is used to develop diagnostic skills for malaria parasite detection.

Both the sensitivity (83.2%) and specificity (90.1%) by the microscopists in this study were lower than the sensitivity and specificity of other studies, respectively, 96.8% and 96.7% in Ethiopia [26], 88% and 91% in Zambia [30]. The sensitivity but not the specificity of this study was relatively higher than a sensitivity of 83% and specificity of 97% by a study conducted in Bahirdar, Ethiopia [28], and sensitivity of 63% and specificity of 95.7% in Mekelle, Ethiopia [29]. The lower sensitivity than specificity in the current study on detection of parasites indicates that there was relatively a high rate of false negative results, which means that misdiagnosis of true infections was high.

Overall, strength of agreement in identification of different species of malaria in the current study (K: 0.11) was lower than that of a study conducted in Ethiopia where k: 0.33 [26], in North Gondar where K: 0.47 [31] and Hawassa town with k: 0.63 [27]. Low performance on species identification may be due to the fact that some participants did not spend sufficient time to examine the slides or possibly due to lack of regular training. The current study participants were from geographies with low malaria endemicity which were targeted for malaria elimination, and this condition might make the microscopists to have low exposure or frequency to examine malaria cases which inturn lowering the diagnostic capacity. Lack of correct species identification may lead to incorrect administration of first-line treatment. For example, the recommended first-line treatment for uncomplicated *P*. *falciparum* malaria in Ethiopia is artemether-lumefantrine (AL) while the first-line treatment for *P*. *vivax* is oral chloroquine [32]. Correct species identification is used to treat an individual with an appropriate first-line drug and used to prevent drug resistance resulting due to incorrect drug administration.

In the current study, the false positivity rate (negative BF slides reported as positive among all positive reports by participants) was 2.9% which was lower than the finding of a study conducted in Hawassa town where the rate was 6.9% [27], 12% report in Hawassa, Ethiopia [33], and 19% report in Democratic Republic of Congo [34], but higher than the finding of a study conducted in Ethiopia where the rate was 0.8% [26], and 2.0% report in Canada [35]. These false positive results could lead to unnecessary treatment with anti-malarial drug or a delayed diagnosis of the true cause of illness and misleading the clinician from considering other causes of fever and disease. The false negativity rate (positive slides reported as negative per all negative reports by participants) in our study was 42.7% which was higher than 11.5% report in Ethiopia [26], 10.1% report in Democratic Republic of Congo [34]. False negativity can lead to delayed treatment, development of serious complications and possibly death or exposure to unnecessary treatment with other non anti-malarial drugs.

About 230 (97.0%) of the participants used plus system of parasite estimation or screening which is less accurate and non-recommended method per the current WHO guideline. Those who estimated the paraite density by plus system (97%) in the current study were higher than those who estimated parasites by screening in a study conducted in Ethiopia (31.8%) [26], and in Democratic Republic of Congo where 68.6% of the participants used this quantification system [34]. Parasite quantification using non-recommended system may be due to lack of updated information (lack of training), or lack of awareness of the advantage of the quantitative system over a semi quantitative (plus) system, or it might be due to lack of commitment to adhere to specified time for counting parasites with the recommended quantification system. Knowing exact estimate of parasites can be used to monitor patient response to treatment and to study drug efficacy; if the drug is efficient and there is a progress in patient response to treatment the parasite density will decrease frequently from the baseline parasite count. However, the current study showed failure of the participants to count parasites which inturn undermining the profession of microscopists not to be involved in malaria examination for measuring treatment response and/or therapeutic efficacy.

## Conclusion

Most of the supplies which are important for malaria laboratory diagnosis using microscope were not available or insufficient in some of the study health facilities.The findings also showed gaps in laboratory set up, documentation and Giemsa solution mainly at health centers and private clinics than district hospitals. Malaria microscopists in the current study achieved good grade in parasite detection and poor grade in parasite species identification. The findings showed that the microscopists were “*In-Training*” level specially by species identification and parasire counting. The low skill of malaria microscopists with poor laboratory set up, lack of documentation, and poor quality of supplies & equipment may be an obstacle for malaria elimination in the study settings. Therefore, Federal Ministry of Health and Ethiopian Public Health Institute in collaboration with regional health bureaus and partners should conduct regular on-site supervision, check for staff competency, try to fulfill the shortages in reagents and equipment in the laboratory, prepare important documents and records and provide comprehensive *In-service* training. Certifying laboratory professionals led by the WHO country office & Ministry of Health should be commenced to have gold standard microscopy service. This study was not addressed some areas of the country which have moderate and high malaria transmission. So we recommend future studies to be conducted through out the country which includes all epidemiological strata.

## Supporting information

S1 File Annexes. (DOCX)

## Abbreviations

API: Annual parasite incidence
EPHI: Ethiopian Public Health Institute
EQA: External Quality Assurance
HCs: Health Centers
FEN: Foundation for Emerging Nations
IFCC: International Federation of Clinical Chemistry
NSP: National Strategic Plan
PC: Private Clinics
RDT: Rapid Diagnostic Tests
SOP: Standard Operating Procedures and WHO: World Health Organization.
