# Supplementary material for: Comprehensive competency assessment of malaria microscopists and laboratory diagnostic service capacity in districts stratified for malaria elimination in Ethiopia​
Source: PLoS One. 2020 Jun 25;15(6):e0235151. doi: 10.1371/journal.pone.0235151 (PMC7316265; doi:10.1371/journal.pone.0235151)
Supplement: S1 File — (DOCX) [file pone.0235151.s001.docx]

# ANNEXES

## ANNEX 1: GENERAL QUESTIONS FOR MALARIA MICROSCOPISTS

Facility Name: ___________________________________District_________________________

Zone____________________________________Region______________________________

Participant Name: ________________________ID:___________ Date: _____________

Profession: Lab technician Lab technologist Phone: _____________________

| **No** | **Questions** | **Response Category** | **Remark** |
| --- | --- | --- | --- |
|  | Age in Years | _______________ |  |
|  | Sex | 1. Male 2. Female |  |
|  | When did you Graduate? | 1. Less than 1yr 2. 1-2 years 3. >2 years (_________yrs) |  |
|  | Which college did you graduate from? | 1. Government 2. Private 3. Other_______ |  |
|  | What is your qualification? | 1. Diploma 2. Bachelor degree 3. Master’s degree 4. other_____________ |  |
|  | Field of study/s: | | |
|  | What course did you take in your college concerning malaria diagnosis? | 1. Theoretical only 2. Theoretical & Practical |  |
|  | How long have you worked as malaria microscopist? | _______________ |  |
|  | Have you taken In-service training on malaria microscopy? | 1. Yes 2. No |  |
|  | If yes, when and how many times? | 1. Once: ______(date) 2. 2 times:______,_______ 3. 3-times______,_____,______ 4. More: _______,______________ |  |
|  | When was your last training? | ______________________________ |  |
|  | Who did give you training? | 1. Government:__________________________________ 2. NGO:__________________ |  |
|  | Do you diagnose malaria detection? | 1. Yes 2. No |  |
|  | Do you identify malaria species? | 1. Yes 2. No |  |
|  | Do you identify all parasite life stages? | 1. Yes 2. No |  |
|  | Do you perform parasitaemia count? | 1. Yes 2. no |  |
|  | If yes, which count do you use? | 1. +, ++, +++, ++++ 2. Parasite per micro liter/ WBC 3. Parasite per micro liter/ RBC 4. % infected RBC |  |

## ANNEX 2: FACILITY INFORMATION QUESTIONNAIRE

Form for assessment of malaria microscopy laboratories

| Name of laboratory or facility: | | | Date of visit |
| --- | --- | --- | --- |
|  |  |  | (dd/mm/yyyy):___/___/_______ |
| Type of facility: |  Rural clinic/HC |  District hospital |  Zonal hospital |
|  |  Private hospital |  Private Laboratory |  Other (specify)_______ |
| Physical address of laboratory: | | | |
| District, city, town: | | | Zone: |
| Telephone: | | Fax: | E-mail: |
| Name of head of department or director of laboratory: | | | |
| Name of head of facility: | | | |
| **Names of microscopist interviewed: Education & degree**  **1._________________________________________ _______________________________________**  **2.__________________________________________ ________________________________________**  **3._________________________________________ ________________________________________**  **4.__________________________________________ ________________________________________**  **5.__________________________________________ ________________________________________** | | | |
| **Name of interviewer:** | | | |

**General malaria diagnostic service**

| **Questions** | **Response**  **Category** | **Remark** |
| --- | --- | --- |
| Is the lab registered and licensed to practice microscopy (for private laboratory)? | 1. Yes 2. No |  |
| Is there any supervision from regional or national laboratories? | 1. Yes 2. No |  |
| Does your lab participate in EQA programs? | 1. yes 2. no |  |
| Which do you use, microscope or RDT? | 1. Microscope 2. RDT 3. other_________ |  |
| Is the lab provide routine malaria smear microscopy service | 1. yes 2. no |  |
| How many malaria slides do you examine daily? | 1. Less than 5 2. From 5-10 3. More than 10, if so______ |  |
| Is the staining solution are accessible and stored in appropriate place | 1. Yes 2. No |  |
| Where do you get the Giemsa stock from? | 1. Regional lab 2. Facility purchasing  3. EPHI 4. NGOs: -------------------------------------------------------------------  5. Other:------------------------------------- |  |
|  |  |  |
| Do you have properly functional microscope? | 1. Yes 2. No |  |
| Which malaria blood smear do you use? | 1. Thin film only 2. Thick film only   3. Thick and thin film |  |
| Do you perform parasitaemia count? | 1. Yes 2. No |  |
| If yes, which methods do you use | 1. +, ++, +++, ++++ 2. Parasite/µl/WBC 3. Parasite/µl/RBC |  |
| Is there a regular training program for microscopists? | 1. Yes 2. No |  |

**Documentation**

| Questions | Response category | Remark |
| --- | --- | --- |
| Are pathology request forms available? | 1. Yes 2. No |  |
| Are results recorded in an organized and legible manner in logbooks? | 1. Yes 2. No |  |
| Are approved SOPs available in the laboratory? | 1. Yes 2. No |  |
| Are technical manuals and bench aids available in the laboratory? | 1. Yes 2. No |  |
| Are internal QC log sheets available? | 1. Yes 2. No |  |
| Are maintenance logbooks for microscopes and pH meters available? | 1. Yes 2. No |  |

**Laboratory Procedures**

1. **Blood film preparation**

|  | Yes | No | Remarks |
| --- | --- | --- | --- |
| Are SOPs available for blood film preparation? |  |  |  |
| Are both thick and thin films prepared? |  |  |  |
| Are blood films labeled appropriately? |  |  |  |
| Is the quality of prepared blood films monitored? |  |  |  |
| Are unstained slides protected from insects and Auto- fixation? |  |  |  |

1. **Blood film staining**

| Questions | Response  category | Remark |
| --- | --- | --- |
| Are SOPs available for blood film staining? | 1. Yes 2. No |  |
| Are recommended reagent preparation procedures followed? | 1. Yes 2. No |  |
| Is internal QC performed regularly with known positive and negative slides during staining? | 1. Yes 2. No |  |
| What is the staining technique used? | 1. Giemsa stain 2. Other(specify)___________ |  |
| Is buffered distilled water pH 7.2 ± 0.2 used to dilute the Giemsa stain? | 1. Yes 2. No |  |
| Is the Giemsa working stain solution freshly prepared before each staining (within 4 hr)? | 1. Yes 2. No |  |

1. **Blood film examination**

|  | Yes | No | Remarks |
| --- | --- | --- | --- |
| Are SOPs available for examination of blood films? |  |  |  |
| Do microscopists routinely report the presence or absence of parasites, species and density? |  |  |  |
| Do microscopists report parasite density in the WHO-recommended way (parasites/µL)? |  |  |  |
| Does the workload allow the recommended reading time (at least 10 min) per slide? |  |  |  |
| Are examined slides stored and archived properly? |  |  |  |
|  | Fill out dashes | | |
| Average number of slides read per month |  | | |
| No. of slides read per day |  | | |
| Average no. of slides read per day per microscopist |  | | |
| No. of slides archived or assessed by validator |  | | |

**Quality assurance**

|  | Yes | No | Remarks |
| --- | --- | --- | --- |
| Does the laboratory comply fully with the national QA and QC guidelines? |  |  |  |
| Is there a formal protocol for analyzing internal QC results and taking corrective action if the results are not satisfactory? |  |  |  |
| Does the laboratory or microscopists regularly participate in a proficiency testing scheme or other form of external QA? |  |  |  |
| Is the performance of the laboratory or microscopists in the proficiency testing scheme or other form of external quality assessment satisfactory? |  |  |  |
| Does the laboratory have procedures to address poor performance in proficiency testing or other forms of external quality assessment? |  |  |  |

**Laboratory set-up and environment**

|  | **Good** | **Poor** | **Remarks** |
| --- | --- | --- | --- |
| Bench space |  |  |  |
| Sink, washing area, staining area |  |  |  |
| Access to clean water supply |  |  |  |
| Natural lighting |  |  |  |
| Power source |  |  |  |
| Ventilation |  |  |  |
| Storage space for supplies and materials |  |  |  |
| Storage space for unstained and examined slides |  |  |  |
| Secure storage space for confidentiality of patient results |  |  |  |

Good: at least 2-bench space, chair for clients and for lab professionals, windows designed for ventilation, store etc

**Bio-safety**

|  | Yes | No | Remarks |
| --- | --- | --- | --- |
| Laboratory staff wear protective laboratory coats and gowns and safety glasses |  |  | No pockets, with tight cuffs |
| Staff wear gloves when collecting and handling blood samples |  |  |  |
| Hand-washing facilities with soap (or similar) available |  |  |  |
| Power supply for the microscope(s) and laboratory lighting in good condition and safe |  |  |  |
| Containers for dry waste |  |  |  |
| Containers for infectious materials |  |  |  |
| Puncture-resistant container for sharps and blood slides |  |  |  |
| Disposal of waste materials as per national guidelines |  |  |  |

**Equipment and reagents**

- ***Microscope***

|  | **Yes** | **No** | **Remarks** |
| --- | --- | --- | --- |
| The microscope(s) is binocular with oil immersion x100 objective |  |  |  |
| The microscope lamp(s) has sufficient power to provide good illumination when the condenser aperture is set at the correct setting for the x100 objective |  |  |  |
| Blood films can be brought into sharpfocus at x100 oil immersion magnification. |  |  |  |
| The stage movement mechanism is precise and stable. |  |  |  |
| The microscope is placed on a stable bench, with adequate working space and away from staining areas and vibration producing equipment such as centrifuges |  |  |  |
| The microscope(s) is regularly serviced. |  |  |  |
| The microscope(s) is cleaned and protected with a cover after use. |  |  |  |
| Xylene is used to clean the microscope(s), objectives or eye pieces |  |  | Xylene is carcinogenic and should not be used. |
| Spare microscope bulbs are available. |  |  |  |

- ***Microscopic slides***

|  | **Yes** | **No** | **Remark** |
| --- | --- | --- | --- |
| Microscope slides are of good quality and are thoroughly cleaned before use. |  |  |  |
| Microscope slides do not have scratches or surface aberrations. |  |  |  |
| Microscope slides do not give a blue background color after staining. |  |  |  |
| Microscope slides do not have fungal contamination. |  |  |  |
| Microscope slides that have been damaged by fungus are discarded and not used again. |  |  |  |
| In areas with high humidity, microscope slides are protected against fungal contamination. |  |  |  |
| Microscope slides are re-used. |  |  |  |

- ***Staining reagents***

|  | **Yes** | **No** | **Remark** |
| --- | --- | --- | --- |
| All required staining reagents are available. |  |  |  |
| All staining reagents are within the recommended expiry date. |  |  |  |
| Staining solutions are stored as per the manufacturer’s recommendations. |  |  |  |
| SOPs are available for preparation of working stain solutions. |  |  |  |
| Internal QC is performed for each batch of working stain solution prepared and each batch of commercially prepared stain opened for use. |  |  |  |
| Commercial stain solutions do not contain excessive stain precipitate. |  |  |  |
| The cap of the reagent bottle is always tightly sealed except when stain is being removed for use. |  |  |  |
| Stain is always removed from the reagent bottle with a clean pipette or similar. |  |  |  |
| Water is never added to the stock stain solution. |  |  |  |
| Unused stain is never returned to the stock bottle. |  |  |  |
| All required staining reagents are available. |  |  |  |

- ***General laboratory supplies***

|  | Presence | | | Remarks |
| --- | --- | --- | --- | --- |
|  | No | Sufficient | Insufficient |  |
| Alcohol and cotton (or similar) for cleaning skin prior to blood collection |  |  |  |  |
| Lancets |  |  |  |  |
| Methanol |  |  |  |  |
| Giemsa stain |  |  |  |  |
| Buffer salts or buffer tablets |  |  |  |  |
| pH meter accurate to two decimal places |  |  |  |  |
| pH calibration solutions |  |  |  |  |
| Staining jar |  |  |  |  |
| Microscope light bulbs |  |  |  |  |
| Spreader (for making blood films) |  |  |  |  |
| Laboratory gowns |  |  |  |  |
| Safety glasses |  |  |  | including over-spectacles type |
| Gloves, disposable |  |  |  |  |
| Lens cleaning solution |  |  |  |  |
| Marker pens |  |  |  |  |
| Sharps containers |  |  |  |  |
| Needles and syringes |  |  |  |  |
| Vacuum vein puncture supplies |  |  |  |  |
| Pencils, grease, red, glass-writing |  |  |  |  |
| Slide labels |  |  |  |  |
| Cover slips |  |  |  |  |
| Mounting medium |  |  |  |  |
| Tourniquet |  |  |  |  |
| Wound cover strips |  |  |  |  |
| Staining rack |  |  |  |  |
| Drying rack |  |  |  |  |
| Graduated cylinders of the correct size |  |  |  |  |
| Wash bottles |  |  |  |  |
| Timers, sufficient number for staining for each microscopist |  |  |  |  |
| Immersion oil of acceptable viscosity (not too thick and not too thin) |  |  |  |  |
| Tally counters, sufficient number for the number of staff |  |  |  |  |
| Lens paper |  |  |  |  |
| Slide boxes for storage |  |  |  |  |
| For laboratories in which stain is prepared from powder: sufficient glycerol, methanol, powder, beakers, measuring cylinders, filter paper, funnels, stirringrods, scales, spatulas and storage bottles |  |  |  |  |

**Performance indicators**

| Monitoring of: | Yes | No | Remarks |
| --- | --- | --- | --- |
| Total number of slides examined |  |  |  |
| Total number of positive slides, stratified by species |  |  |  |
| Consumption of commodities |  |  |  |
| Monthly stock-outs of microscopy reagents |  |  |  |
| Turnaround time for microscopy results |  |  |  |

**General findings and recommendations:**

**-------------------------------------------------------------------------------------------------------**

**-------------------------------------------------------------------------------------------------------**

**-------------------------------------------------------------------------------------------------------**

**Supervisor or auditor’s comments:**

-------------------------------------------------------------------------------------------------------

-------------------------------------------------------------------------------------------------------

-------------------------------------------------------------------------------------------------------

**Accomplished by:**

**Name of data collector ________________________________________________________**

**Signature _____________________________________________________**

**Date:** -----------------------------------------------------
